# Full-Space Three-Dimensional Holograms Enabled by a Reflection–Transmission Integrated Reconfigurable Metasurface

**DOI:** 10.3390/nano15141120

**Published:** 2025-07-18

**Authors:** Rui Feng, Yaokai Yu, Dongyang Wu, Qiulin Tan, Shah Nawaz Burokur

**Affiliations:** 1 State key Laboratory of Extreme Environment Optoelectronic Dynamic Measurement Technology and Instrument, North University of China, Taiyuan 030051, China; ruifeng@nuc.edu.cn (R.F.); sz202319046@st.nuc.edu.cn (Y.Y.); sz202206135@st.nuc.edu.cn (D.W.); 2 Key Laboratory of Micro/Nano Devices and Systems, Ministry of Education, North University of China, Taiyuan 030051, China; 3 LEME, Univ Paris Nanterre, F92410 Ville d’Avray, France

**Keywords:** reconfigurable metasurface, hologram, full-space, reflection–transmission, microwave

## Abstract

A metasurface capable of flexibly manipulating electromagnetic waves to realize holograms presents significant potential in millimeter-wave imaging systems and data storage domains. In this study, full-space three-dimensional holograms are realized from a reflection–transmission integrated reconfigurable metasurface, which can achieve nearly 360° phase coverage in reflection space and 180° phase coverage in transmission space. By adjusting the voltage applied to the constituting electronically tunable meta-atoms of the metasurface, an octahedron hologram constituted by three hologram images in different focal planes is generated in the reflection space at 6.25 GHz. Moreover, a diamond hologram, also composed of three hologram images in different focal planes, is achieved in the transmission space at 6.75 GHz. Both the numerical simulation and experimental measurement are performed to validate the full-space holograms implemented by the modified weighted Gerchberg–Saxton (WGS) algorithm with specific phase distribution in different imaging planes. The obtained results pave the way for a wide range of new applications, such as next-generation three-dimensional displays for immersive viewing experiences, high-capacity optical communication systems with enhanced data encoding capabilities, and ultra-secure anti-counterfeiting solutions that are extremely difficult to replicate.

## 1. Introduction

Holography was first invented by Dennis Gabor in 1948 as a method to create three-dimensional object images by recording the interference patterns of object and reference waves [1]. Though the development of laser technology in 1960 significantly advanced holographic imaging in optics, enabling arbitrary beam shaping and predefined image reconstruction [2,3], traditional holography faced limitations such as reliance on coherent light sources, speckle noise, and high requirements for optical materials. In response, incoherent holography technologies emerged, including zone-plate coded holography proposed by Mertz and Young [4], as well as Fresnel incoherent correlation holography (FINCH) and coded aperture correlation holography (COACH) developed by Rosen et al. [5,6], which eliminated the need for coherent light and improved imaging performance. In 1966, Brown and Lohmann introduced computer-generated holography (CGH), which generates holograms through diffraction calculations, expanding design freedom to realize virtual images [7]. However, conventional phase holograms require light to propagate over distances far longer than the wavelength to accumulate enough phase variation for wavefront shaping. Due to the limited refractive index of natural materials, the optical elements for such holograms generally show large unit cell sizes.

Metasurfaces [8,9,10,11,12], planar artificial metamaterials composed of subwavelength-scale two-dimensional (2D) elements referred to as meta-atoms, have the ability to manipulate electromagnetic (EM) waves by a judicious arrangement of the constituting meta-atoms. By designing discontinuous phase shifts, metasurfaces can flexibly tailor impinging EM wavefronts, enabling novel phenomena and applications such as anomalous refraction/reflection [13,14,15], metalenses [16,17,18], metasurface-based operators [19,20,21,22], polarization conversion [23,24,25,26,27], and orbital angular momentum (OAM) wave generation [28,29,30,31], to name a few. Compared to traditional 3D metamaterials [32,33,34], they offer advantages such as lower profile, easier fabrication, and greater flexibility in electromagnetic (EM) wave control. Owing to the unprecedented capability of wavefront control at the subwavelength scale, metasurfaces are capable of achieving holographic imaging with high spatial resolution while eliminating unwanted diffraction orders. While the functionality in traditional passive metasurfaces is fixed, loading metasurfaces with electronic components showing tunable features enables programmable functionalities [35]. Hence, a tunable metasurface has enabled to achieve programmable holography where the holograms can be dynamically tailored in real time [36]. However, such reconfigurable metasurfaces are generally based on the 1-bit coding scheme for reflection, transmission, or full-space hologram generation.

In this paper, full-space three-dimensional (3D) hologram is achieved utilizing a reflection–transmission integrated reconfigurable metasurface, which can realize quasi-360° phase coverage in reflection space and 180° phase coverage in transmission space. By dynamically adjusting the bias voltage applied to the varactor diodes embedded in the constituting meta-atoms of the metasurface, the phase response of the metasurface can be reconfigured in real time, enabling the generation of 3D holograms in both reflection and transmission domains. Specifically, an octahedron hologram composed of three distinct focal patterns (single spot at *z* = −20 mm, four spots at *z* = −85 mm, and single spot at *z* = −150 mm) is demonstrated in the reflection space at 6.25 GHz, while a diamond-shaped hologram with six spots at *z* = 55 mm, six spots at *z* = 85 mm, and a single spot at *z* = 150 mm is realized in the transmission space at 6.75 GHz.

## 2. Metasurface Design

**Figure 1:**
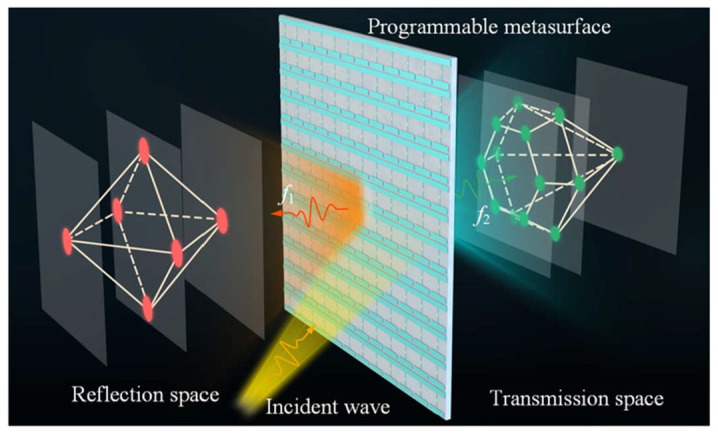
Schematic illustration of the 3D holograms realized using the reflection–transmission integrated reconfigurable metasurface. Under a unidirectional incident wave, two holograms are produced at different frequencies and in different spaces.

**Figure 2:**
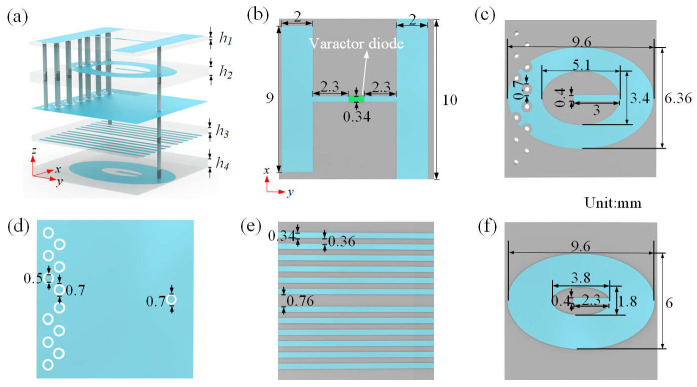
Topological configuration of the proposed reflection–transmission integrated reconfigurable unit cell. (**a**) Three-dimensional view of the unit cell. (**b**) Top layer. (**c**) Second layer. (**d**) Third layer. (**e**) Fourth layer. (**f**) Fifth layer. All dimensions are in mm.

A schematic of the full-space hologram utilizing the reflection–transmission integrated reconfigurable metasurface is shown in Figure 1, where the hexahedron image is generated in the reflection space and the diamond image is achieved in the transmission space with a unidirectional illumination by adjusting the bias voltages applied to the metasurface in real time. It is worthwhile to note that full-space wave manipulation can also be achieved from Janus metasurfaces [37,38], where different electromagnetic responses are tailored upon bidirectional illuminations, i.e., both forward and backward illuminations. The configuration of the designed unit cell is presented in Figure 2. As illustrated by the 3D view in Figure 2a, the unit cell is composed of five metallic layers interspaced by four dielectric substrate layers having a relative permittivity of *ε*ᵣ = 3.55 and a loss tangent of tan *δ* = 0.001. The thicknesses of the four dielectric substrates are *h*_1_ = 0.3 mm, *h*_2_ = 0.9 mm, *h*_3_ = 0.6 mm, and *h*_4_ = 0.9 mm, respectively. The top layer incorporates two parallel copper strips and an embedded varactor diode of model MACOM MAVR-011020-1411, as shown in Figure 2b. One of the strips is continuous in the *x* direction and connected to the negative sign (ground) of the DC voltage supply, while the other one is connected to the positive sign of the DC voltage supply through the connecting metal via and feed line printed on the fourth layer. The second receiving layer used for the transmission function is an elliptical patch integrating a U-slot in the center, as displayed in Figure 2c. The third layer is a metallic reflection plane with 13 ring-shaped slots to isolate the metal vias and avoid short-circuits, as presented in Figure 2d. The fourth layer is composed of 12 feed lines, as illustrated in Figure 2e, which are connected to the positive sign of the DC voltage. The fifth radiating layer features a U-slot-loaded elliptical patch, as in the second layer but with different geometric dimensions, as shown in Figure 2f. One strip of the top layer, the second receiving layer, and the fifth radiating layer are connected by one metal via a hole to the ground.

**Figure 3:**
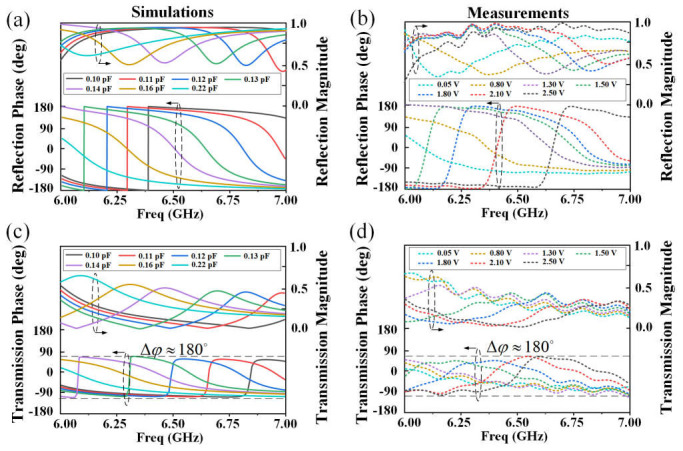
Simulated and measured responses of the unit cell in the frequency band spanning from 6 GHz to 7 GHz for different capacitance values of the varactor diode and applied bias voltage. (**a**) Simulated reflection responses. (**b**) Measured reflection responses. (**c**) Simulated transmission responses. (**d**) Measured transmission responses.

To evaluate the electromagnetic performances of the proposed unit cell, numerical simulations are performed using the commercial ANSYS HFSS software. Periodic boundary conditions are applied to the unit cell in the *x*- and *y*-directions under a *y*-polarized wave incidence. A Floquet port serves as the plane wave source to illuminate the structure as well as to record the reflection response, while a second one serves as a listening port to record the transmission response. Figure 3 presents the simulated and measured responses of the unit cell for both reflection and transmission in the frequency band spanning from 6 GHz to 7 GHz. The varactor embedded in the unit cell has a dynamic capacitance range of 0.02–0.22 pF, and seven capacitance values are selected to highlight the responses. As shown in Figure 3a,b, the reflection phase covers nearly 360° in both simulations and experiments. The simulated and measured transmission responses are displayed in Figure 3c,d, where the phase achieves a 180° coverage, thus meeting the requirements of a 1-bit phase coding scheme. It should be noted that due to the use of the commercial varactor diode featuring an intrinsic resistance, a portion of the incident power is absorbed. The discrepancy between simulation and measurement results is predominantly attributed to manufacturing tolerances of the reconfigurable metasurface. Here, the unit cell is designed by empirical formulas and optimizations through parameter tuning. However, it should be noted that the design speed of the unit cell and voltage control schemes can be greatly improved using artificial intelligence or machine learning techniques [39,40].

## 3. Principles and Results

To achieve the proposed holographic imaging, the enhanced weighted Gerchberg–Saxton (WGS) algorithm is leveraged to derive the required phase profiles [41,42,43]. This methodology involves modeling ideal point sources as virtual emitters positioned at specified hotspot coordinates. In contrast to the conventional GS algorithm, a tuning parameter is introduced to strike an optimal balance between holographic imaging efficiency and predefined intensity ratio across target focal points. Notably, the electromagnetic wave propagation in this scenario deviates from the paraxial approximation due to the focal length being on the same order of magnitude as the operational wavelength. Consequently, the propagation model adopted in the enhanced WGS algorithm for this design explicitly incorporates Green’s function formulation [44].

Let us consider a system comprising *N* hotspots distributed in the imaging plane and *M* meta-atoms that constitute the metasurface structure. The phase delay *φ_m_* imposed by the *m*th meta-atom (*m* = 1, 2, …, *M*) is determined by superposing electromagnetic fields generated by all *n* virtual sources (*n* = 1, 2, …, *N*), with each field interaction described via Green’s function formalism. Through this superposition principle, the electromagnetic field converging to the *N* target hotspots is reconstructed from the collective scattering of all *M* meta-atoms. The iterative procedure for optimizing the desired phase profiles can thus be formalized as follows [45]:

(1) $$\varphi_{m}^{p}=arg\left(\sum_{n=1}^{N}\frac{e^{ikr_{m}^{n}}}{r_{m}^{n}}\frac{w_{n}^{p}E_{n}^{p-1}}{\left|E_{n}^{p-1}\right|}\right)$$

(2) $$E_{n}^{p}=\sum_{m=1}^{M}\frac{e^{-ikr_{m}^{n}+i\varphi_{m}^{p}}}{r_{m}^{n}}$$

(3) $$w_{n}^{p}=w_{n}^{p-1}\frac{\sum_{n=1}^{N}\left|E_{n}^{p-1}\right|}{\left|E_{n}^{p-1}\right|}\frac{q_{n}}{\sum_{n=1}^{N}q_{n}}$$

where $r_{m}^{n}$ represents the distance between the *n*th hotspot and the *m*th meta-atom, with the superscript *p* indicating the iteration number. *E_n_* denotes the electric field intensity of the *n*th hotspot, and *w_n_* serves as a weight factor that governs the energy ratio among the *n* hotspots. The parameter *q_n_* allows one to specify the intensity ratio of the *n*th hotspot relative to the others. The initial values of the weight factor *w* and the phase term *φ* are set as follows:

(4) $$w_{n}^{0}=1,\varphi_{m}^{0}=\frac{2\pi m}{M}$$

To facilitate a quantitative evaluation of the quality of the generated holographic images, the imaging efficiency and total efficiency for both reflection and transmission holograms are calculated. The imaging efficiency can be defined as follows:

(5) $$\eta_{image}=\frac{P_{image}}{P_{detect}}$$

where $P_{image}$ is defined as the power conveyed by the image, and $P_{detect}$ represents the reflected or transmitted power within the detection plane. Considering the losses of the metasurface, the total efficiency is then expressed as follows:

(6) $$\eta_{total}=\frac{P_{image}}{P_{inc}}$$

where *P_inc_* denotes the power incident on the metasurface.

**Figure 4:**
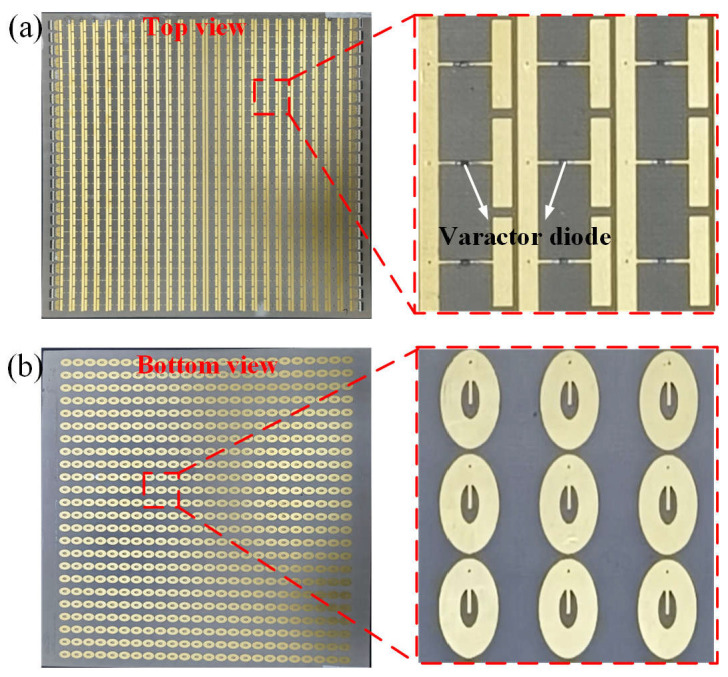
Photographs of transmission–reflection integrated reconfigurable metasurface. (**a**) Top view of the metasurface. (**b**) Bottom view of the metasurface.

**Figure 5:**
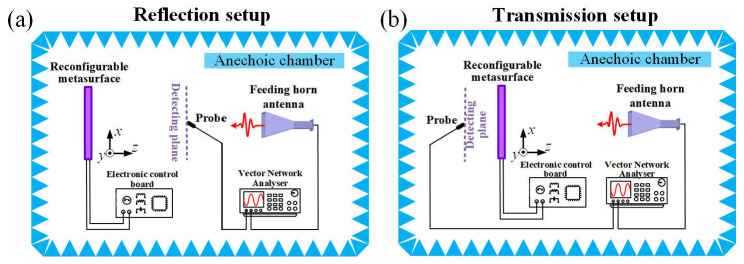
Schematic illustration of the experimental measurement setups. (**a**) Schematic setup for the electric field measurement in the reflection space. (**b**) Schematic setup for the electric field measurement in the transmission space.

Based on the designed meta-atom, a programmable metasurface platform is developed, comprising an array of 24 × 24 unit cells with an overall dimension of 240 × 240 mm^2^. As illustrated in Figure 4a,b, the metasurface prototype is fabricated via a standard printed circuit board (PCB) fabrication process. The varactor diodes are fixed to the metasurface using surface-mount technology (SMT). To experimentally validate the realization of the holograms in both reflection and transmission spaces, measurements are performed in a microwave anechoic chamber, as illustrated by the near-field scanning benches shown in Figure 5a,b. A 2–18 GHz broadband horn antenna, situated at a sufficiently large distance from the metasurface, is employed as the excitation source to generate linearly polarized quasi-plane waves. An electric field waveguide probe is mounted on a computer-controlled two-axis linear positioning system. This configuration allows for a two-dimensional mapping of the electric field within a maximum scanning area of 400 × 400 mm^2^. Both the horn antenna and waveguide probe are interfaced to the two ports of a vector network analyzer to facilitate signal generation and data acquisition.

**Figure 6:**
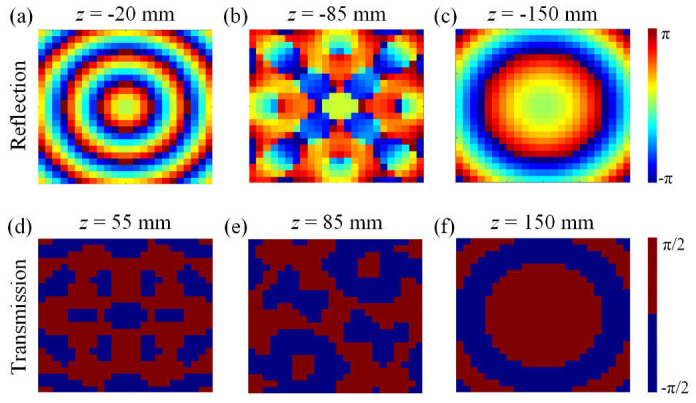
Phase profiles of the holograms in reflection space at (**a**) *z* = −20 mm, (**b**) *z* = −85 mm, and (**c**) *z* = −150 mm. Phase profiles of the holograms in transmission space at (**d**) *z* = 55 mm, (**e**) *z* = 85 mm, and (**f**) *z* = 150 mm.

The enhanced WGS algorithm, incorporating Green’s function to address non-paraxial propagation, is used to derive phase profiles for full-space holography. Figure 6 displays the calculated phase distributions at different distances for both the octahedron hologram in the reflection space (Figure 6a–c) and for the diamond hologram in transmission space (Figure 6d–f). While the image is reconstructed using quasi-360° phase modulation in the reflection space, a 1-bit phase coding scheme is adopted for the image in the transmission space.

**Figure 7:**
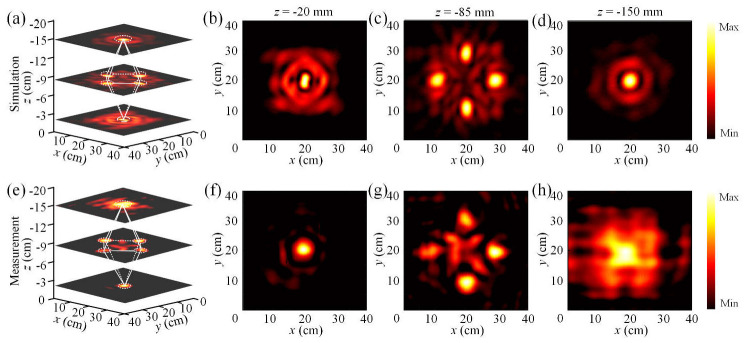
Numerical simulations and experimental measurements of the hologram in reflection space at 6.25 GHz. (**a**) Three-dimensional view of the simulated octahedron hologram. (**b**–**d**) Simulated electric field distributions in the transverse planes at *z* = −20 mm, −85 mm, and −150 mm. (**e**) Three-dimensional view of the measured octahedron hologram. (**f**–**h**) Measured electric field distributions in the transverse planes at *z* = −20 mm, −85 mm, and −150 mm.

To reconstruct the hologram in the reflection space using the designed reconfigurable metasurface, both the numerical simulations and experimental measurements are performed at 6.25 GHz, and the corresponding results are shown in Figure 7. A 3D view of the simulated octahedron hologram is presented in Figure 7a, where six focusing spots exploited to achieve the octahedron are generated in the reflection space by real-time adjustment of the bias voltages applied to the meta-atoms of the metasurface. The electric field distributions in the different transverse planes at *z* = −20 mm, −85 mm and −150 mm are depicted in Figure 7b–d, respectively. Single central focal points are thus achieved in two outer planes, while four distinct focal points are achieved in the middle inner plane. The corresponding measured results are shown in Figure 7e–h. However, the shapes and intensities of the measured images differ slightly from the simulated ones, which can be attributed to the imperfections of the metasurface sample and also to the measurement environment. Furthermore, according to Equations (5) and (6), the simulated imaging and total efficiencies of the four focal spots in the plane *z* = −85 mm are calculated as 51.85% and 36.99%, while the corresponding measured performances are calculated to be 46.26% and 27.83%, respectively.

**Figure 8:**
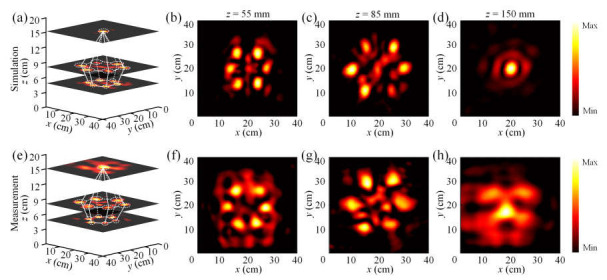
Numerical simulations and experimental measurements of the hologram in transmission space at 6.75 GHz. (**a**) Three-dimensional view of the simulated diamond hologram. (**b**–**d**) Simulated electric field distributions in the transverse planes at *z* = 55 mm, 85 mm, and 150 mm. (**e**) Three-dimensional view of the measured diamond hologram. (**f**–**h**) Measured electric field distributions in the transverse planes at *z* = 55 mm, 85 mm, and 150 mm.

Numerical simulations and experimental measurements are carried out at 6.75 GHz to validate the generation of the hologram in the transmission space, and the relevant results are displayed in Figure 8. As illustrated by the 3D view of the diamond hologram in Figure 8a,e, imaging spots in three different planes are adopted to create the 3D hologram. As such, six differently spaced focusing points are generated in two different planes (*z* = 55 mm and *z* = 85 mm), and a single focusing point is generated in the *z* = 150 mm transverse plane. The measurement outcomes shown in Figure 8e–h demonstrate qualitative consistency with the simulation results. In addition, the simulated imaging efficiency and total efficiency of the six focal spots in the plane *z* = 85 mm are calculated as 42.79% and 21.17%, while the corresponding measured imaging efficiency and overall efficiency of the same six focal spots at *z* = 85 mm are calculated to be 36.24% and 18.74%, respectively. The measured efficiencies are lower than in simulations, which is mainly due to the ohmic losses introduced by the electronic components. The inevitable processing tolerances for the metasurface fabrication, the quasi-plane wave rather than ideal plane wave incidence, and possible slight misalignment of the illuminating horn antenna are all possible factors that can negatively affect the experimental imaging quality.

## 4. Conclusions

In this study, we have successfully demonstrated the realization of full-space 3D holography using a reflection–transmission integrated reconfigurable metasurface. The metasurface achieves nearly 360° phase coverage in reflection and 180° in transmission, enabling versatile wavefront manipulation. By implementing the enhanced weighted Gerchberg–Saxton algorithm, we have generated an octahedron hologram in reflection space at 6.25 GHz and a diamond hologram in transmission space at 6.75 GHz by exploiting three different imaging planes so as to reconstruct the 3D image. The proposed design approach combines the advantages of reconfigurable metasurfaces with full-space holography, paving the way for applications requiring dynamic 3D visualization and multi-dimensional electromagnetic wave control. In addition, such a full-space hologram based on the reflection–transmission integrated reconfigurable metasurface concept can be further transposed to the terahertz (THz) frequency band by replacing the varactor diode with a tunable component in the THz range, such as semiconductors, phase-change materials, graphene, and other suitable 2D materials [46]. The THz full-space metasurface shows potential for integration with upcoming 6G and ultrafast communication technologies. Moreover, the proposed reconfigurable metasurface can be further exploited in programmable diffractive deep neural networks (D2NNs), which have potential applications in real-time adaptive beamforming, bidirectional optical computing, and multispectral sensing.

## Data Availability

Data is contained within the article.

## Abbreviations

The following abbreviations are used in this manuscript:

WGS	Weighted Gerchberg–Saxton
FINCH	Fresnel incoherent correlation holography
COACH	Coded aperture correlation holography
CGH	Computer-generated holography
EM	Electromagnetic
OAM	Orbital angular momentum
3D	Three-dimensional
PCB	Printed circuit board
SMT	Surface-mount technology
